# Joint effect of sleep duration and sleep quality on self-rated health among Canadian adults: estimating relative excess risk due to interaction from a nationwide survey

**DOI:** 10.3389/fpubh.2025.1632239

**Published:** 2025-09-17

**Authors:** Shirmin Bintay Kader, Nahin Shakurun, Jubayer Mumin, Naomi Noor

**Affiliations:** ^1^Department of Community Health and Epidemiology, College of Medicine, University of Saskatchewan, Saskatoon, SK, Canada; ^2^Department of Global Public Health, Karolinska Institutet (KI), Solna, Sweden

**Keywords:** self rated health, sleep quality, trouble sleeping, CCHS, sleep health, RERI

## Abstract

**Background:**

Self-rated health (SRH) is a globally recognized measure of health status. Previous studies have established that inadequate sleep duration and trouble falling asleep combinedly has a greater negative impact on health than either factor alone. This study aims to investigate the excess relative risk due to the interaction between short sleep duration and trouble sleeping on SRH.

**Method:**

We used the 2017–18 Canadian Community Health Survey (CCHS) publicly used microdata file. SRH was measured on a 5-point Likert scale from poor to excellent and dichotomized into “Good or Better” and “Fair or Poor.” Sleep duration was categorized into “Less than 7 h” and “More than 7 h,” while trouble sleeping was categorized as “Yes” or “No.” A joint variable derived from these created four groups: “no sleep issues,” “fewer sleeping hours (<7 h) only,” “trouble sleeping only,” and “fewer hours & trouble sleeping.” A weighted univariable and multivariable logistic regression with robust variance estimation was conducted to estimate relative risk due to interaction.

**Results:**

Among Canadian adults, 40.96% reported less than 7 h of sleep, and 48.38% reported trouble sleeping. Approximately 11% had Fair or Poor SRH. The odds ratio for Fair or Poor SRH was 1.34 (95% CI: 1.23–1.46) for short sleep duration, 2.38 (95% CI: 2.18–2.61) for troubled sleep, and 2.97 (95% CI, 2.66–3.33) for both conditions. The adjusted RERI was 0.80 (95% CI, 0.40–1.21).

**Implication:**

These results imply that shorter duration of sleep and troubled sleeping may increase the negative influence on self-rated health.

## 1 Background

Sleep is essential for maintaining health and enhancing overall well-being (1). Previous studies have highlighted the importance of adequate sleep- both duration and quality- for physical and mental health among diverse groups of population around the world (2–4). Multiple studies revealed that insufficient sleep duration significantly increases the risk of obesity, hypertension, coronary heart disease, and stroke among generally healthy populations (5–8). On the other hand, research indicates that sufficient sleep can enhance memory (9) and is linked with self-reported happiness and improved academic achievement (10, 11). A systematic review further revealed that improved sleep quality correlates with numerous positive health outcomes, including better cardiovascular, metabolic, and mental health (12). Kwok et al. (13) conducted a systematic review and reported a divergent result, which revealed that poor quality of sleep and longer duration of sleep had an impact on coronary heart disease and increased moderate risk of mortality, respectively. Nevertheless, many studies highlighted the importance of combined short sleep duration and poor sleep quality, which may increase the likelihood of adverse health outcomes.

Impaired sleep is also associated with poor self-rated health (SRH) (2, 14–18), also known as self-assessed or self-perceived health. SRH is a single-item measure where individuals can evaluate their current health status on a four or five-point scale ranging from excellent to poor. This method is widely used for its reliability and simplicity. Numerous studies across the world have consistently demonstrated that SRH is an effective predictor of mortality from various diseases (19–21). In certain professions such as sports, athletes reported that poor sleep affects not only how they perform, but also how they feel, recover, and even how they define themselves (22). People of all age groups who slept less than 7 h per day reported poor SRH (23, 24). However, adults were more likely to report insufficient sleep. In contrast, Anderson et al. found that among young people, reduced sleep duration exhibited an inverted quadratic relationship with poor SRH, which they attributed to variations in sleep quality (2). Furthermore, Ding et al. (16) conducted a cross-sectional study that revealed that the joint effect of shorter sleep duration and trouble sleeping was much higher than the independent effects of these two sleep variables among medical students.

Similarly, Wang et al. (25) identified that shorter duration of sleep and trouble sleeping had an additive interaction effect on depressive symptoms, indicating that there might be a possibility of the additive effect of trouble sleeping on shorter duration of sleep on SRH. Previously, we conducted a cross-sectional study on the Canadian adult population, where we identified that people with shorter durations and trouble sleeping reported higher poor SRH than those with no sleep issues (14). Although the Odds of poor SRH were reported to be higher among the people with both sleep issues than the individualized odds of shorter sleep and sleep trouble, the additive effect remains unexplored. While previous studies, such as Ding et al. (16) and Wang et al. (25), have examined the joint effects of sleep duration and quality, they differ in outcome focus, study population, and analytical approach. For example, Wang et al. assessed the combined impact of impaired sleep on depressive symptoms among Americans using Relative Excess Risk due to Interaction (RERI), whereas Ding et al. used multivariable-adjusted odds ratios to examine the joint effects of sleep impairment on general health outcomes among Chinese medical students. In contrast, our study uniquely applies an additive interaction framework, specifically RERI, to assess the combined effects of short sleep duration and poor sleep quality on SRH among Canadian adults. This approach enables a more nuanced understanding of potential synergistic effects, which are particularly relevant for informing public health interventions. To the best of our knowledge, this is the first population-based study to investigate the additive interaction between these two sleep-related factors in relation to SRH among Canadian adults using RERI.

## 2 Methods

### 2.1 Study design

This study utilized the publicly available microdata file from the 2017–2018 Canadian Community Health Survey (CCHS) (26). The CCHS is a cross-sectional survey employing a complex multistage design to randomly collect data on sociodemographic and health factors from a nationally representative sample of Canadians aged 12 and older. Data was gathered from all provinces and territories.

### 2.2 Sample size and ethical consideration

The initial survey sample size was 113,290. However, for this study, we focused on a subset of participants with available sleep-related data, totaling 56,675 respondents from Prince Edward Island, Quebec, Alberta, British Columbia, Yukon, and Nunavut. Further restricting the analysis to participants aged 18 and older resulted in a final sample size of 52,378. Given the public availability of the data, this study did not require ethical review.

### 2.3 Study variables

#### 2.3.1 Dependent variable

Self-rated health (SRH) was measured on a five-point Likert scale question, asking participants, “In general, would you say your health is… (Poor, Fair, Good, Very Good, Excellent).” For analysis, we dichotomized SRH into two categories: Good or Better (combining Good, Very Good, and Excellent) and Fair or Poor (combining Fair and Poor).

#### 2.3.2 Independent variables

Two sleep-related variables were selected from the CCHS survey: sleep duration and trouble sleeping. Sleep duration was measured by asking participants to report their average number of hours of sleep per night, which we later categorized as “<7 h” and “≥7 h.” Trouble sleeping was assessed based on the question “How often do you have trouble going to sleep or staying asleep?” and was recoded as a binary variable with “Yes” and “No” responses. Additional sociodemographic variables (age group, sex, ethnicity, marital status, highest education, income, employment status, and immigration status) were also extracted from the CCHS dataset and recoded similarly to the previously published article from the same population (14).

### 2.4 Statistical analysis

Data analysis was conducted by using STATA version 15 (27). We have considered the survey weight during all analyses. To account for the complex sampling design of the dataset, including stratification, clustering, and sample weights, we used the Taylor Series Linearization method to estimate variances and 95% confidence intervals. This was implemented through the ‘svy’ command suite in STATA. A chi-square test with survey weighting was conducted to identify the sociodemographic group difference based on sleeping hours and trouble sleeping. Sample distribution was reported as a weighted percentage, and a significant value was reported if *p* < 0.05. In continuation of the previous study, we followed the procedure to report both unadjusted and adjusted RERI (14) by following the steps below.

### 2.5 Calculating RERI on the additive scale by using stratification

We created a combined variable to analyze RERI based on stratified data. We created a combined variable named “impaired sleep” and categorized it into four groups: No sleep issues (E−M−), Fewer sleeping hours (<7 h) only (E+M−), trouble sleeping only (E−M+), and both fewer sleeping hours & trouble sleeping (E+M+). We used stratification to estimate the RERI, as this approach allows for a direct and interpretable comparison of joint effects across specific combinations of sleep duration and quality categories. This method aligns with the assumptions of additive interaction models and facilitates transparent calculation of stratum-specific risks relative to the reference group. We calculated the RERI based on the following formula to evaluate the value on the additive scale (28).


$$\begin{array}{l} \text{RERI}=\text{OR}\;(E+M+)-\text{OR}\;(E+M-)-\\ \text{OR}\;(E-M+)+1\;(\text{using stratification}). \end{array}$$

Later, we used the “non-linear combination coefficient” (nlcom) command in STATA to calculate the 95% confidence interval (CI) and *p*-value for the reported RERI (29). A calculated RERI greater than “0” indicates supra-additivity with positive interaction on the additive scale (29).

In the final model, we included all significant variables from the univariable logistic regression in a multivariable logistic regression framework, incorporating potential interaction terms to calculate the adjusted RERI on the additive scale by the steps as mentioned above.

## 3 Result

### 3.1 Sociodemographic characteristics of the participants based on sleeping duration and quality

Table 1 shows the weighted percentages of Canadian adults based on sleeping duration and quality. Notably, 40.96 and 48.38% of Canadian adults reported having less than 7 h of sleep and trouble going to sleep or staying asleep, respectively. A higher percentage of individuals reporting less than 7 h of sleep rate their health as “Fair or Poor” (52.69%) compared to those sleeping more than 7 h (47.31%). One-third (32.82%) of the respondents reporting health as “Fair or Poor” experience trouble sleeping compared to those with “Good or Better” health (53.78%), with a significant p-value (<0.001). Age, sex, ethnicity, and employment status showed significant differences across the groups regarding sleep duration and trouble sleeping. Older adults (65 and above) have a higher proportion of sleeping more than 7 h (64.68%) compared to younger age groups; conversely, younger adults (less than 40 years) reported the highest proportion (53.33%) of trouble sleeping. Females (60.34%) are more likely to sleep over 7 h than males (57.71%), whereas men have the highest percentage of trouble sleeping (Male vs. Female: 59.05% vs. 44.37%). While a significantly higher percentage (61.07%) of white Canadian adults reported having slept less than 7 h, the prevalence of having trouble going to sleep among white (51.00%) was slightly lower than among non-white (54.90%) Canadians. Marital status and income show some variation in sleep duration, though not statistically significant. Conversely, respondents who were married (53.07%) and had an income of more than 40 thousand (52.20%) reported a significantly higher percentage of trouble sleeping. There was no significant difference across the education groups in terms of sleep duration and quality. Non-immigrants (60.29%) are also more likely to report less sleep duration compared to immigrants; oppositely, immigrants reported a significantly higher prevalence of trouble sleeping than non-immigrants (55.17 vs. 50.45).

**Table 1:** Distribution of participants’ characteristics (in terms of weighted percentages) across the sleeping issue.

Variables	Number of hours per night usually spent sleeping			Trouble going to sleep or staying asleep		
	<7 h (%) 40.96	≥7 h (%) 59.04	*p*-value	Yes (%) 48.38	No (%) 51.62	*p*-value
Self-rated health (SRH)						
Good or better	59.84 (59.10–60.56)	40.16 (39.44–40.90)	<0.001	53.78 (53.04–54.52)	46.22 (45.48–46.96)	<0.001
Fair or poor	52.69 (50.72–54.75)	47.31 (45.35–49.28)		32.82 (30.98–34.71)	67.18 (65.29–69.02)	
Age group						
<40 years	58.93 (57.71–60.13)	41.07 (39.87–42.29)	<0.001	53.33 (52.10–54.55)	46.67 (45.45–47.90)	<0.001
40–64 years	56.34 (55.24–57.43)	43.66 (42.57–44.76)		49.47 (48.36–50.58)	50.53 (49.42–51.64)	
65 and above	64.68 (63.55–65.79)	35.32 (34.21–36.45)		52.97 (51.77–54.17)	47.03 (45.83–48.23)	
Sex						
Male	57.71 (56.71–58.70)	42.29 (41.30–43.29)	<0.001	59.05 (58.04–60.04)	40.95 (39.96–41.96)	<0.001
Female	60.34 (59.40–61.28)	39.66 (38.72–40.60)		44.37 (43.41–45.34)	55.63 (44.66–56.59)	
Ethnicity						
White	61.07 (60.33–61.81)	38.93 (38.19–39.67)	<0.001	51.00 (50.24–51.76)	49.00 (48.24–49.76)	<0.001
Non-White	52.10 (50.23–53.97)	47.90 (46.03–49.77)		54.90 (53.02–56.77)	45.10 (43.23–46.98)	
Marital status						
Married/common law	59.29 (58.41–60.17)	40.71 (39.83–41.59)	0.06	53.07 (52.17–53.97)	46.93 (46.03–47.83)	<0.001
Windowed/divorced/separated	57.01 (55.54–58.48)	42.99 (41.52–44.46)		47.77 (46.27–49.27)	52.23 (50.73–53.73)	
Single	59.46 (57.99–60.93)	40.54 (39.07–42.01)		49.80 (48.29–51.31)	50.20 (48.69–51.71)	
Highest education						
Less than secondary school	58.66 (56.82–60.47)	41.34 (39.53–43.18)	0.20	50.64 (48.74–52.55)	49.36 (47.45–51.26)	0.54
Secondary school graduate	57.88 (56.39–59.36)	42.12 (40.64–43.61)		51.61 (50.10–53.12)	48.39 (46.88–49.90)	
Post-secondary degree	59.36 (58.50–60.22)	40.64 (39.78–41.50)		51.89 (51.01–52.77)	48.11 (47.23–48.99)	
Income						
Less than 40 thousand	57.94 (56.61–59.27)	42.06 (40.73–43.39)	0.08	49.35 (47.95–50.73)	50.65 (49–27-52.03)	<0.001
More than 40 thousand	59.33 (58.54–60.12)	40.67 (39.88–41.46)		52.20 (51.39–53.01)	47.80 (46.99–48.61)	
Employment status (working status-last 12 month)						
Yes	57.19 (56.33–58.05)	42.81 (41.95–43.67)	<0.001	52.34 (51.48–53.21)	47.66 (46.79–48.52)	<0.001
No	61.94 (60.58–63.28)	38.06 (36.72–39.42)		47.84 (46.46–49.24)	52.16 (50.76–53.54)	
Immigration status						
Non-immigrant	60.29 (59.54–61.04)	39.71 (38.96–40.46)	<0.001	50.45 (49.68–51.23)	49.55 (48.77–50.32)	<0.001
Immigrant	55.12 (53.51–56.72)	44.88 (43.28–46.49)		55.17 (53.54–56.78)	44.83 (43.22–46.46)	

### 3.2 Sleep variables and socio-demographic factors related with poor SRH

Table 2 illustrates the association between sleep duration and trouble sleeping with SRH. Respondents who slept less than 7 h had reported 1.34 times (95% CI: 1.23–1.46, *p* < 0.001) times higher odds for “Fair or Poor” SRH in comparison to the respondents who slept more than 7 h. Similarly, respondents who reported trouble sleeping reported 2.38 times (95% CI: 2.18–2.61, *p* < 0.001) higher odds for “Fair or Poor” SRH in comparison to the respondents who had no trouble issue with their sleep. Table 2 also illustrates that the older age group (65yeras and above OR = 3.76, 95% CI: 3.34–4.23, *p* < 0.01), female (OR = 1.10, 95% CI: 0.82–1.19, *p* < 0.05), unemployment status (OR = 4.11, 95% CI: 3.73–4.52, *p* < 0.001) are significantly related with Fair or Poor SRH. And higher income (OR = 0.40, 95% CI: 0.37–0.44, *p* < 0.001) and with post-secondary education (OR = 0.27, 95% CI: 0.24–0.30, *p* < 0.001) respondents reported 60 and 73% Good or Better SRH, respectively.

**Table 2:** Unadjusted odds ratios with 95% Taylor linearization confidence intervals (OR_unadj_ (95% CI)) for the association of sociodemographic and sleep related factors with SRH, CCSH, 2017–2018, Canada.

Variables	OR_unadj_ (95% CI)	p-value
Age group		
<40 years	1	
40–64 years	2.05 (1.81–2.32)	<0.001
65 and above	3.76 (3.34–4.23)	<0.001
Sex		
Male	1	
Female	1.10 (1.00–1.19)	0.029
Ethnicity		
White	1	
Non-White	0.94 (0.82–1.07)	0.332
Marital status		
Married/common -law	1	
Windowed/divorced/separated	2.21 (2.01–2.43)	<0.001
Single	1.09 (0.97–1.22)	0.142
Highest education		
Less than secondary school	1	
Secondary school graduate	0.40 (0.36–0.46)	<0.001
Post-secondary degree	0.27 (0.24–0.30)	<0.001
Income		
Less than 40 thousand	1	
More than 40 thousand	0.40 (0.37–0.44)	<0.001
Employment (working status-last 12 month)		
Yes	1	
No	4.11 (3.73–4.52)	<0.001
Immigration status		
Non-immigrant	1	
Immigrant	1.01 (0.90–1.12)	0.97
Number of hours per night usually spent sleeping		
Less than 7 h and more than 9 h	1	
In between 7 and 9 h	1.34 (1.23–1.46)	<0.001
Trouble going to sleep or staying asleep		
Yes	1	
No	2.38 (2.18–2.61)	<0.001

### 3.3 The combined effect of fewer sleeping hours and trouble sleeping on SRH

When respondents had fewer sleeping hours (<7 h), the OR for Fair or Poor SRH was 1.14 (95% CI: 0.98–1.33, *p* = 0.09), OR for only trouble sleeping was 1.98 (95% CI: 1.76–2.24, *p* < 0.001). Moreover, the respondents who had faced both fewer sleep and trouble sleeping had reported a 2.97 (95% CI: 2.66–3.33, *p* < 0.001) times higher likelihood of developing Fair or Poor health in comparison to the respondents who had no issue with sleep (Table 3). To assess the access risk of the combined effect of the sleep issue on health, we have conducted the RERI on the additive scale by using the stratified OR’s value from the combined variable, by using nlcom command in STATA and the following formula:


$$\begin{array}{l} {\text{RERI}}_{\text{unadj}}=\text{OR}\;(\begin{array}{l} \text{Combined effect of fewer sleeping}\\ \;\text{hours and trouble sleep} \end{array})-\\ \text{OR}\;(\text{Only fewer sleeping hours})-\\ \text{OR}\;(\text{Only Trouble sleeping})+1. \end{array}$$

=2.97–1.98−1.14+1.

=0.85.

**Table 3:** Unadjusted odds ratios (OR_unadj_) with 95% Taylor linearization confidence intervals (95% CI) for the association between SRH and combined effect (stratified) of fewer sleep and trouble sleep, CCSH, 2017–2018, Canada.

Variable	Good or Better (%)	Fair or Poor (%)	OR_unadj_ (95% CI)	*p*-value
Impaired sleep (IS)				
No sleeping issue	93.75 (93.23–94.23)	6.25 (5.77–6.77)	1	
Fewer sleeping hours	92.93 (92.07–93.70)	7.07 (6.30–7.93)	1.14 (0.98–1.33)	0.085
Trouble sleeping	83.44 (82.40–84.44)	11.67 (10.81–12.59)	1.98 (1.76–2.24)	<0.001
Combined effect of fewer sleep and trouble sleep	89.75 (89.34–90.14)	16.56 (9.86–10.66)	2.97 (2.66–3.33)	<0.001

RERI (from additive scale) 0.85 (95% CI: 0.54–1.17) indicates joint effect of sleep variables shows a positive supra-additive interaction on Fair or Poor SRH.

Based on the results from univariable logistic regression and the associations observed in Table 1, we identified potential confounders for inclusion in the final model examining the relationship between impaired sleep and SRH. After adjusting for potential confounders (age, sex, marital status, education, income, and working status), in final analysis, we found that individuals who reported fewer than 7 h of sleep had 1.31 times higher odds of reporting Fair or Poor SRH (95% CI: 1.09–1.57, *p* = 0.003) (Table 4). Those who experienced only trouble sleeping had 2.24 times higher odds (95% CI: 1.94–2.58, *p* < 0.001). Notably, respondents reporting both short sleep duration and trouble sleeping had a 3.35-fold higher likelihood (95% CI: 2.94–3.83, *p* < 0.001) of fair or poor SRH compared to those without any sleep issues (Table 4, Supplementary Table 1). From this final model (by using nlcom command in STATA and formula below):


$$\begin{array}{l} {\text{RERI}}_{\mathrm{adj}}=\text{OR}\;(\begin{array}{l} \text{Combined effect of fewer sleeping}\\ \;\text{hours and trouble sleep} \end{array})-\\ \text{OR}\;(\text{Only fewer sleeping hours})-\\ \text{OR}\;(\text{Only Trouble sleeping})+1. \end{array}$$

=3.35–2.24−1.31+1.

=0.80.

**Table 4:** Adjusted odds ratios (OR_adj_) with 95% Taylor linearization confidence intervals (95% CI) for the association between SRH and combined effect (stratified) of fewer sleep and trouble sleep, CCSH, 2017–2018, Canada.

Number of hours per night usually spent sleeping	Trouble going to sleep or staying asleep	
	No	Yes
No sleep issue	Ref	2.24 (1.94–2.58) **
>7 h	1.31 (1.09–1.57)	3.35 (2.94–3.83)**

** Significant at *p* < 0.001.

The RERI value of 0.80 (95% CI: 0.40–1.21), calculated on the additive scale after adjusting for confounders, also indicates a positive supra-additive interaction between sleep duration and sleep trouble on fair or poor self-rated health (SRH). From this final model, the adjusted RERI value further confirmed a synergistic effect of the combined sleep issues on SRH.

## 4 Discussion

This study explored the combined effects of short sleep duration (<7 h) and trouble sleeping on SRH among Canadian adults, utilizing data from the 2017–2018 Canadian Community Health Survey. The results revealed significant additive interactions between these sleep disturbances, emphasizing their compounded negative impact on SRH.

We first assessed if socio-demographic factors (age, sex, and socio-economic status) altered the link between impaired sleep and self-rated health (SRH). No significant modification was found, so the final model only adjusted for these variables as potential confounders. After removing non-significant interaction terms, the adjusted RERI remained positive (RERI_adj_ = 0.80; 95% CI: 0.40–1.21), indicating a possible supra-additive effect, meaning the combined impact of short sleep and sleep difficulty might be greater than the sum of their individual effects. However, because the lower confidence interval bound is close to zero, there is some uncertainty, and the joint effect could partly be due to unmeasured or residual confounding.

The relationship between sleep disturbances and SRH has been extensively studied, consistently demonstrating that both short sleep duration (24) and poor sleep quality (30, 31) are independently associated with adverse health outcomes. Additionally, Seow et al. (32) reported independent and combined associations of sleep duration and sleep quality with common physical and mental disorders.

Our findings reinforce previous observations, indicating that Canadian adults who both experience short sleep duration (<7 h) and encounter difficulties sleeping are nearly three times more likely to report Fair or Poor SRH than those without sleep problems. This is consistent with the research conducted by Ding and colleagues (16) which found that medical students with short sleep duration and poor sleep quality also had higher odds of reporting suboptimal SRH, which is further evidenced by a recent systematic review (33). However, another systematic review (2) reported that poor sleep quality was associated with worse SRH in long sleep duration but not short sleep duration.

The combined effects of reduced sleep duration and difficulties with sleep can be scientifically elucidated through their impact on mental health, particularly in relation to depression. Research has demonstrated that sleep deprivation leads to neurobiological changes, including a reduction in dopamine receptor availability in the striatum, which negatively affects mood regulation and heightens the risk of depression (34). Additionally, chronic sleep deprivation disrupts the hypothalamic–pituitary–adrenal (HPA) axis, resulting in increased cortisol levels and heightened stress responses (35). This physiological stress can exacerbate emotional dysregulation, further elevating the likelihood of developing depressive symptoms (25).

Sleep disruption is associated with increased activity of the sympathetic nervous system and the hypothalamic–pituitary–adrenal axis, metabolic effects, changes in circadian rhythms, and proinflammatory responses. In otherwise healthy adults, short-term consequences of sleep disruption include increased stress responsivity, somatic pain, reduced quality of life, emotional distress, mood disorders, and cognitive, memory, and performance deficits. Long-term consequences of sleep disruption in otherwise healthy individuals include hypertension, dyslipidaemia, cardiovascular disease, weight-related issues, metabolic syndrome, type-2 diabetes mellitus, and colorectal cancer (36). Additionally, all-cause mortality is increased in men with sleep disturbances (36, 37). The combined effect may accelerate health deterioration, as indicated by the supra-additive risk identified in the study.

From a public health perspective, the high prevalence of short sleep duration (40.96%) and trouble sleeping (48.38%) among Canadian adults suggests that a substantial portion of the population is at elevated risk for Fair or Poor SRH due to these combined sleep disturbances. Targeted interventions, such as sleep hygiene education and cognitive-behavioral therapy for insomnia (CBT-I), are essential to mitigate these risks and enhance overall health outcomes (38).

This study has several strengths, including its utilization of large, comprehensive, and representative population-based data. However, certain regions such as Nunavut and Yukon are overrepresented, while others are excluded, which may limit the generalizability of our findings to all Canadian adults across provinces and territories. Nonetheless, this study provides substantial evidence on additive effect of sleep duration and sleep quality on SRH among Canadian adults. These findings can inform policymakers and healthcare professionals in planning and developing different intervention approaches to reduce sleep disturbance and improve SRH among diverse population groups in Canada. Clinically, our results highlight the importance of routinely assessing both sleep duration and quality during patient evaluations, as their combined impact may significantly affect overall health perceptions and wellbeing. Healthcare providers should be aware that patients presenting with poor sleep quality and short sleep duration are at increased risk for poor health outcomes, which could warrant early intervention or referral to sleep specialists.

It is important to note that, the cross-sectional nature of the survey data, the causal relationship could not be established. Moreover, our study focused exclusively on short sleep duration (<7 h) in combination with poor sleep quality and did not account for long sleep duration (>9 h), which has also been shown in previous research to adversely affect self-rated health. Besides, this study relies on secondary administrative data, which does not include information related with potential confounders, such as medication use. As a result, we were unable to account for the potential effects of medications on sleep quality and overall quality of life, which may confound the observed relationships between impaired sleep and SRH. This study also did not account for certain confounders such as comorbidities, obesity, medication use, and alcohol intake, which may influence sleep and health outcomes. Future studies should explore the full spectrum of sleep duration to better understand its joint effects with sleep quality on health outcomes.

Our study provides valuable insights on joint effect of sleep duration and quality on SRH of Canadian adults. The nearly threefold increase in Fair or Poor SRH among those experiencing both short sleep duration and sleep difficulties aligns with some previous research, though conflicting evidence exists. These findings underscore the complex interplay between sleep and health, suggesting that both sleep duration and quality should be considered in health assessments and interventions. Future longitudinal research with qualitative component is needed to fully understand these relationships across different populations and contexts.

## Data Availability

Publicly available datasets were analyzed in this study. This data can be found here: https://www150.statcan.gc.ca/n1/daily-quotidien/191022/dq191022d-eng.htm.
