# Supplementary material for: Joint effect of sleep duration and sleep quality on self-rated health among Canadian adults: estimating relative excess risk due to interaction from a nationwide survey
Source: Front Public Health. 2025 Sep 17;13:1632239. doi: 10.3389/fpubh.2025.1632239 (PMC12483862; doi:10.3389/fpubh.2025.1632239)
Supplement: Supplementary file 1 [file Table_1.DOCX]

**Supplementary Table 1: Adjusted Odds ratios with 95 % Taylor Linearization confidence intervals (OR_adj_ (95% CI) for the association of sociodemographic and impaired sleep with SRH, CCSH, 2017–2018, Canada**

| **Variables** | **OR_adj_ (95% CI)** | **p-value** |
| --- | --- | --- |
| **Impaired sleep (IS)** |  |  |
| No sleeping issue | 1 |  |
| Fewer sleeping hours | 1.31 (1.09-1.57) | 0.003 |
| Trouble sleeping | 2.24 (1.94-2.58) | <0.001 |
| Combined effect of fewer sleep and trouble sleep | 3.35 (2.94-3.83) | <0.001 |
|  |  |  |
| **Age group** |  |  |
| <40 years | 1 |  |
| 40-64 | 1.92 (1.68-2.19) | <0.001 |
| 65 & above | 1.36 (1.15-1.60) | <0.001 |
| **Sex** |  |  |
| Male | 1 |  |
| Female | 0.87 (0.79-0.97) | 0.012 |
| **Marital Status** |  |  |
| Married/common law | 1 |  |
| Windowed/divorced/separated | 1.41 (1.24-1.59) | <0.001 |
| Single | 1.31 (1.15-1.51) | <0.001 |
| **Highest Education** |  |  |
| Less than secondary school | 1 |  |
| Secondary school graduate | 0.59 (0.51-0.69) | <0.001 |
| Post-secondary degree | 0.43 (0.37-0.49) | <0.001 |
|  |  |  |
| **Income** |  |  |
| Less than 40 thousand | 1 |  |
| More than 40 thousand | 0.64 (0.57-0.71) | <0.001 |
| **Employment (Working status-last 12 month)** |  |  |
| Yes | 1 |  |
| No | 3.12 (2.76-3.53) | <0.001 |
